# Effects of Different Marker Set Configurations on Tennis Stroke Recognition Performance: A Three-Dimensional Kinematic Study Based on MiniRocket

**DOI:** 10.3390/s26165081

**Published:** 2026-08-11

**Authors:** Qiang Xu, Dian Jiao, Yuanwu Zhu, Yiqing Wang, Yunchao Ma

**Affiliations:** 1 College of P.E. and Sports, Beijing Normal University, Beijing 100875, China; 202522070046@mail.bnu.edu.cn (Q.X.); 202522070015@mail.bnu.edu.cn (D.J.); 202632070022@mail.bnu.edu.cn (Y.Z.); 2 College of Systems and Society, Australian National University, Canberra, ACT 2601, Australia

**Keywords:** tennis, three-dimensional motion capture, machine learning, MiniRocket, action recognition, marker-set configuration

## Abstract

Three-dimensional motion capture provides high-fidelity kinematic trajectories, but the extent to which different marker configurations retain discriminative information for tennis stroke recognition remains unclear. This study combined 3D motion capture with MiniRocket to compare seven upper-limb and racket marker configurations using data from 40 tennis-trained participants, 13 stroke types, and 10,133 valid movement samples. The configurations were the full-information group (ALL), full-arm group (AB), forearm group (FA), forearm + racket group (FR), upper-arm group (UA), racket group (RK), and watch group (WT). Model performance was evaluated using leave-one-subject-out cross-validation. All configurations achieved high performance in the three-class task. In the 13-class task, Macro-F1 was highest for ALL (78.76%), followed closely by FR (78.71%) and RK (78.28%). Holm-adjusted pairwise comparisons showed no significant differences among ALL, FR, and RK. FR significantly outperformed AB, FA, UA, and WT, whereas RK significantly outperformed FA, UA, and WT. Serve and overhead strokes were easiest to recognize, while several fine-grained baseline and net-play strokes showed lower F1-scores. These findings indicate that discriminative information is more strongly represented in the forearm-wrist-racket chain rather than simply increasing with marker count.

## 1. Introduction

Three-dimensional (3D) motion capture can accurately record the spatiotemporal changes in human movement and is an important tool in sports biomechanics [1]. Research on tennis strokes based on 3D marker trajectories has gradually expanded from the computation of conventional variables such as joint angles, joint moments and joint angular velocities to the use of spatiotemporal trajectory features as inputs for machine-learning algorithms in action recognition and movement-pattern analysis [2]. In parallel, advances in artificial intelligence have provided new analytical pathways for action recognition: machine-learning methods can automatically extract patterns from complex time-series data and improve the efficiency of large-scale movement-data processing while maintaining relatively low computational demands [3,4,5]. These developments make the automatic recognition of tennis strokes from 3D motion-capture data feasible and provide technical support for training feedback and performance evaluation [6,7,8].

Existing research on tennis stroke recognition has followed several technical routes. Video-based methods use RGB frames, optical flow or human skeleton information together with models such as support vector machines (SVM), convolutional neural networks (CNN) or long short-term memory networks (LSTM) to recognize forehand, backhand, serve and volley strokes [9,10]; however, video approaches are sensitive to camera viewpoint, occlusion and background variation, and have a limited ability to resolve the high-speed motion of the racket and subtle differences in upper-limb trajectories. Wearable inertial measurement unit (IMU) methods record local inertial signals using accelerometers and gyroscopes mounted on the wrist or the racket and classify strokes with random forests, temporal convolutional models and similar approaches [11,12,13,14]; nevertheless, a single sensor mainly reflects motion information at one local site and cannot fully reconstruct the 3D spatial relationships between body segments and the racket [15]. By comparison, methods based on 3D motion-capture data provide high-accuracy trajectory information for both the whole body and the implement, and recent studies have used graph neural networks such as ST-GCN and A3T-GCN to recognize strokes in datasets such as 3DTennisDS [16,17,18]. However, these studies were conducted on independent datasets under different experimental conditions and focused mainly on improving classifier accuracy, whereas the input side of 3D motion-capture experiments—that is, how the marker-set configuration itself affects recognition performance—has not been systematically compared. In sports biomechanics, the number and placement of markers directly affect the quality of kinematic data and the complexity of experimental procedures, and more markers do not necessarily yield a more stable or more effective movement representation [19,20,21,22]. For tennis stroke recognition, the contribution of different anatomical regions to discriminative information remains unclear, and which marker configurations can preserve sufficient discriminative information while reducing the number of channels still requires systematic investigation.

To address this gap, the present study combines 3D motion capture with the MiniRocket time-series classification model to systematically compare seven upper-limb and racket marker configurations for tennis stroke recognition. The main innovations and contributions are as follows: (1) in a 3D motion-capture dataset comprising 40 participants, 13 stroke classes and 10,133 valid trials were constructed; (2) under relatively strict validation conditions, the recognition performance of seven marker configurations was systematically compared, providing empirical evidence for the optimization of marker sets; and (3) systematic evidence on the contribution of marker configuration to stroke recognition was obtained, providing a theoretical basis for selecting the key sensor placements for stroke recognition in wearable devices.

The remainder of this paper is organized as follows. Section 2 reviews related work. Section 3 describes the materials and methods in detail. Section 4 presents the results. Section 5 discusses the findings. Section 6 addresses the limitations and future perspectives. Section 7 concludes the paper.

## 2. Related Work

### 2.1. Techniques and Methods for Tennis Stroke Recognition

Sports action recognition aims to automatically detect and distinguish the specific movements performed by athletes using sensor, image or movement-trajectory data. The data sources used in existing studies mainly include video and images, inertial measurement units (IMUs), depth sensors, human skeleton data and optical 3D motion capture. Early studies typically extracted hand-crafted time-domain, frequency-domain or kinematic features and then applied traditional machine-learning methods such as support vector machines (SVMs), k-nearest neighbours, decision trees and random forests. With the development of deep learning, convolutional neural networks (CNNs), recurrent neural networks (RNNs), long short-term memory networks (LSTMs), graph convolutional networks (GCNs) and Transformers have gradually been used to learn the spatial and temporal features of movements. Related studies indicate that supervised learning has remained the dominant modelling approach in sport-specific action recognition; SVM was particularly widely used in earlier IMU and vision studies, whereas deep models have further advanced end-to-end spatiotemporal feature learning [9,15,23,24].

Video-based tennis action recognition is non-contact, convenient to deploy, and well suited to match settings. Early studies mainly used visual features such as player silhouettes, optical flow and histograms of oriented gradients, combined with hidden Markov models, SVM or conditional random fields, to recognize forehand, backhand, serve and non-stroke actions. With advances in artificial intelligence, researchers began to use RGB images, depth maps and human skeleton data to recognize more fine-grained tennis strokes, successively adopting SVM, linear-chain conditional random fields, CNN and LSTM. A representative approach extracts single-frame spatial features with a pre-trained CNN and then models the historical information between consecutive frames with a multi-layer LSTM, thereby recognizing different types of forehand, backhand, volley, serve and overhead strokes [3,4,9]. However, video methods are easily affected by camera viewpoint, background variation, player occlusion, and the small apparent size of the racket in long-distance images; for strokes with small local differences, such as flat, topspin and slice strokes, appearance features alone may not stably capture fine kinematic information.

Wearable sensors provide another technical route for tennis stroke recognition. IMUs usually integrate accelerometers and gyroscopes and can record the linear accelerations and angular velocities of a local body segment or of the racket during a stroke. Previous studies have mounted sensors on the racket-holding wrist, on the racket or at other body sites and have distinguished forehand, backhand, serve and different spin types by signal segmentation, filtering and hand-crafted feature extraction combined with SVM, k-nearest neighbours, random forests, neural networks or temporal convolutional models. Ebner and Findling further compared the wrist and the racket as sensor positions and showed that the mounting position changes the movement information recorded by the sensor and the resulting classification performance [25]. Wearable solutions offer relatively low cost, good portability and convenient real-time recognition, but a single sensor mainly reflects local inertial changes and cannot directly recover the complete 3D spatial relationship between body segments and the racket; model performance may also be affected by sensor placement, orientation deviations and individual stroke style [16,17,18].

With the development of depth cameras and optical motion-capture technology, 3D skeleton or marker trajectories have begun to be used directly for tennis stroke recognition. A 3D skeleton sequence can be represented as the spatial coordinates of multiple joints over time, and the corresponding deep-learning methods mainly include RNN, CNN, GCN and Transformer. Among these, GCN can construct a graph structure according to the connections between human joints or markers and can learn spatial topology and temporal variation simultaneously, and is therefore well suited to human movement trajectories [24]. In tennis, THETIS uses Kinect to obtain markerless skeleton data; Tennis-MoCap uses an optical marker-based system to record body movement and stores it in BVH format; and 3DTennisDS uses a Vicon system to collect the 3D trajectories of both the body and the racket and verifies the action-recognition capability of the data with ST-GCN [26,27,28]. A subsequent study on 3DTennisDS used 39 body markers and seven racket markers as input to an A3T-GCN and showed that adding racket information to whole-body trajectories improved the recognition of forehand, backhand and volley strokes, indicating that implement trajectories are an important source of information in racket-sport recognition [29].

Existing studies of tennis action recognition have mainly focused on the choice of data modality, the form of feature representation and the improvement of classification models. Video studies emphasize visual representations and spatiotemporal networks, wearable studies focus on local inertial signals and their practical deployment, and 3D motion-capture studies emphasize the spatial accuracy and topological relationships of body and racket trajectories. By contrast, studies that systematically compare marker configurations of different anatomical regions and of the racket under identical data, identical classifiers and identical cross-subject validation remain limited. Three-dimensional motion-capture data are essentially multivariate time series composed of multiple coordinate channels; therefore, in addition to deep neural networks, dedicated time-series classification methods can also be applied. MiniRocket converts time series into features using a set of almost deterministic convolutional kernels and completes recognition with a linear classifier; it is computationally fast and has low stochasticity, which makes it suitable for repeated training across multiple marker configurations and multiple LOSO folds [30]. Compared with ROCKET, MiniRocket replaces random sampling with fixed kernel lengths, dilations and biases and is therefore faster and almost deterministic; compared with deep models such as InceptionTime, ResNet and Transformer, its training and inference costs are markedly lower. Accordingly, the aim of the present study is not to demonstrate that MiniRocket outperforms all deep-learning models, but to use MiniRocket, under a consistent classification method and validation procedure, to compare the discriminative information preserved by different marker configurations.

### 2.2. Overview of Public Tennis Action Recognition Datasets

Public datasets provide a common data basis for the development, reproduction and comparison of action-recognition algorithms. At present, the most representative public tennis action-recognition datasets are THETIS, Tennis-MoCap and 3DTennisDS. They use a Kinect depth camera, an OptiTrack marker-based motion-capture system and a Vicon marker-based motion-capture system, respectively, and represent different data-collection routes ranging from video and low-resolution skeleton information to high-accuracy 3D body and racket trajectories. Because they differ in acquisition equipment, participant sample size, action categories, data format and whether racket information is included, these datasets are suited to different action-recognition tasks but also have their own limitations. A comparison of the key characteristics of the three representative datasets is shown in Table 1.

THETIS is an early and widely used public tennis action-recognition dataset. It uses a Microsoft Kinect to record 12 classes of tennis strokes performed by 55 participants, comprising 8374 action videos. Its stroke classes include different types of forehand, backhand, volley, serve and overhead strokes. With a relatively large number of participants, a comparatively rich set of stroke classes and multiple data modalities, it is suitable for video-based action recognition, depth-information analysis and skeleton-sequence classification, and it has been used to develop and validate models including support vector machines, conditional random fields, convolutional neural networks and long short-term memory networks.

Tennis-MoCap uses six OptiTrack Flex V100 infrared cameras to record the 3D movements of 17 tennis players at a sampling frequency of 100 Hz. The dataset includes serve, overhead, forehand groundstroke, forehand volley, backhand groundstroke and backhand volley strokes, and 106 BVH motion files are publicly available. During acquisition, 34 markers were placed on the body, and the exported BVH data represent the human skeleton with 23 3D points of interest. Compared with THETIS, Tennis-MoCap obtains more explicit 3D human movement information through a marker-based optical system and reduces the influence of skeleton-estimation errors of depth cameras on trajectory analysis.

3DTennisDS uses eight Vicon cameras to record forehand, backhand, forehand volley and backhand volley strokes performed by 10 professional tennis players at a sampling frequency of 100 Hz, comprising 769 stroke trials. The dataset uses the Plug-in Gait model with 39 retro-reflective markers on the body and seven markers on the racket, and the data are stored in C3D format. Compared with the two datasets above, 3DTennisDS provides high-accuracy 3D trajectories of both the body and the racket, which allows the role of racket information in action recognition to be studied more directly and is also suitable for models such as ST-GCN and A3T-GCN that analyze the spatial topology and temporal variation among markers.

Overall, existing public datasets already cover video, markerless skeletons, marker-based human skeletons and joint body–racket 3D trajectories. However, datasets that simultaneously provide high-accuracy racket trajectories, a large participant sample and fine-grained stroke classes are still lacking; more importantly, no study has yet performed a controlled comparison of marker configurations across different body regions and the racket under identical data, identical classification methods and identical cross-subject validation.

No acquisition system is optimal for all tasks. Video and Kinect systems are non-contact, convenient to deploy and relatively low in cost, and are suitable for large-scale acquisition in real training or match environments. Marker-based optical systems such as OptiTrack and Vicon require a dedicated laboratory space, marker placement and considerable data post-processing, and may also be affected by marker occlusion. By contrast, Vicon can directly record the high-frequency 3D position changes in anatomical markers and racket markers under controlled experimental conditions and avoids part of the error introduced by image-based pose estimation. It is therefore particularly suitable for analyzing subtle trajectory differences among the wrist, forearm, upper arm and racket, and for examining the influence of different marker configurations on action recognition. The suitability of Vicon in the present study follows from the requirements of the research question with respect to 3D trajectory accuracy and the controllability of marker placement. In addition, because this study focuses on the relative differences among marker configurations, and because all configurations were derived from the same acquisition system and the same set of trials, the accuracy level of the acquisition system itself does not act as a confounding factor in the comparison among configurations.

On the basis of the above research gap, the present study combines high-accuracy 3D motion-capture data with the MiniRocket machine-learning algorithm to examine the relationship between marker configuration and tennis stroke recognition performance. This design aims to provide a basis for simplifying marker-based motion-capture protocols and to propose experimentally supported candidate regions for sensor placement in wearable devices.

## 3. Materials and Methods

### 3.1. Participants

The required sample size was estimated with G*Power (v3.1.9.2, University of Düsseldorf, Germany) for a two-tailed paired-samples *t*-test, assuming a medium effect size of dz = 0.5, α = 0.05 and statistical power of 80%, which indicated that at least 34 participants were required. Forty participants with a tennis-specific training background were finally recruited. The participants represented intermediate-level recreational tennis players with stable training experience; neither beginners nor elite professional players were included. The inclusion criteria were: (i) right-hand dominance; (ii) stable tennis stroke experience with an NTRP rating above 3.0 (most participants were rated 3.0–3.5 and a small number 4.0); (iii) no acute upper-limb injury within the previous three months; and (iv) no high-intensity exercise within 48 h before the experiment. Descriptive statistics of the participants were as follows: age 25.29 ± 5.18 years, height 174.44 ± 7.89 cm and body mass 69.62 ± 9.13 kg. All participants signed an informed consent form before the experiment. The study was approved by the Ethics Committee of the School of Physical Education and Sports, Beijing Normal University (approval no. TY20260307).

### 3.2. Experimental Equipment and Marker Configurations

**Figure 1:**
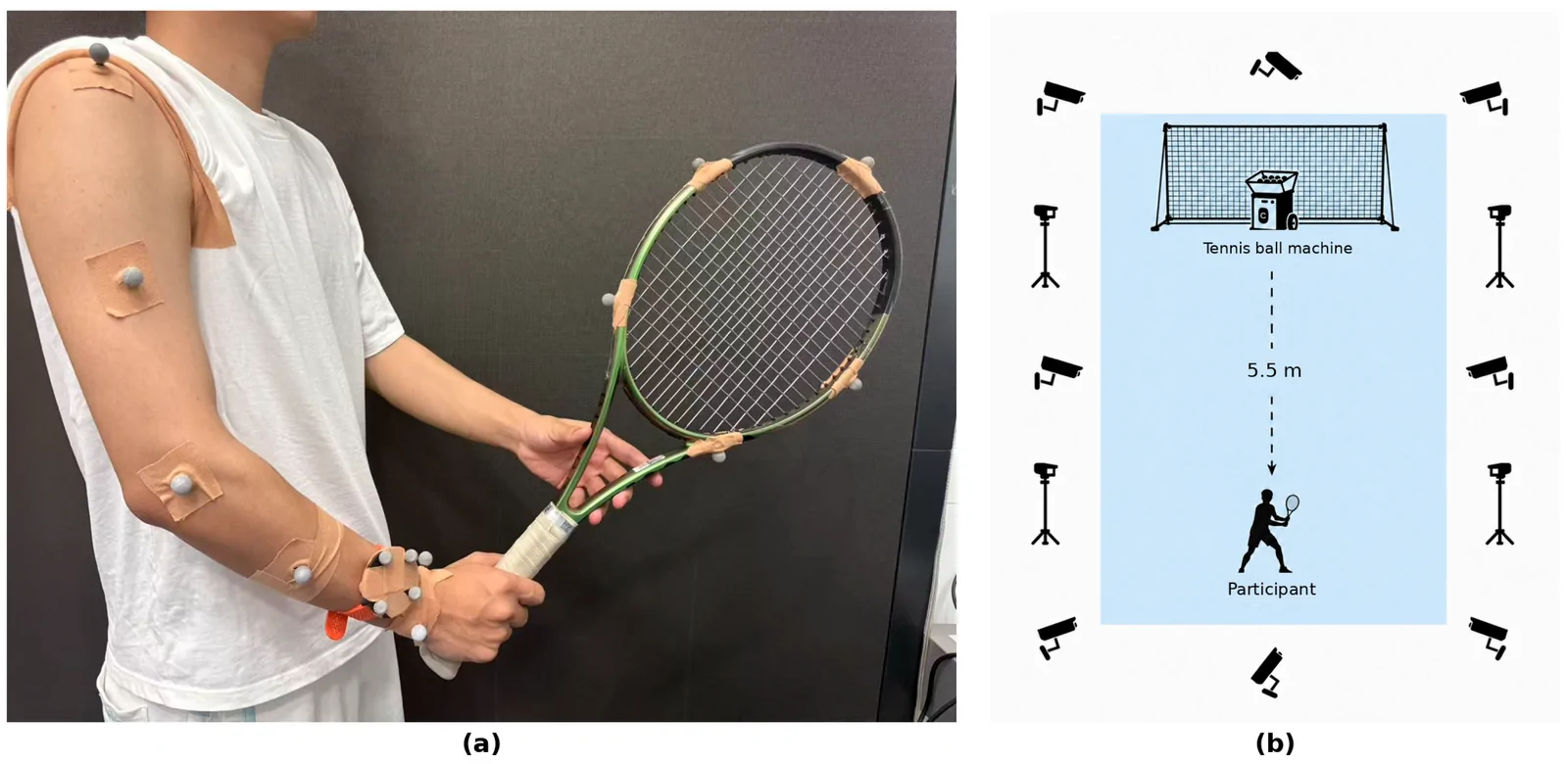
Marker placement and laboratory set-up. (**a**) Photograph of the retro-reflective markers attached to the dominant upper limb and the racket. (**b**) Schematic diagram of the laboratory set-up.

A Vicon T40 three-dimensional motion-capture system consisting of 12 high-speed infrared cameras (sampling frequency 200 Hz; Vicon Motion Systems Ltd., Oxford, UK) was used to record the 3D coordinate trajectories of markers on the upper limb and the racket. The laboratory set-up and marker placement are shown in Figure 1. For every participant, the same investigator attached 16 retro-reflective markers with a diameter of 14 mm to the dominant upper limb and to the racket. The same racket (head size 98 in^2^, mass 295 g, string tension 50 lbs) and the same ball machine (Jbotsports JT-M5, Shanghai Jingwang Sporting Goods Co., Ltd., Shanghai, China) were used throughout the experiment. Participants were positioned approximately 5.5 m from the ball machine, a distance close to the 5.485 m between the service line and the baseline in tennis, which corresponds to the flight distance of the ball after it bounces in real play. A trial was regarded as a valid stroke when the participant hit the ball over the net into the court. Data pre-processing, model training, validation and testing were performed with a Python-based machine-learning framework (Python 3.11) on a computer running 64-bit Windows 11 with an AMD Ryzen 7 5800 H processor and an NVIDIA GeForce RTX 3060 graphics processing unit (GPU). The corresponding software environment included sktime 0.40.1, scikit-learn 1.7.2, NumPy 2.3.5, pandas 2.3.3, SciPy 1.17.1, Numba 0.62.1, joblib 1.5.3 and openpyxl 3.1.5. Generative artificial intelligence was used as an auxiliary tool at several stages of this work, and all of its output was verified by the authors. ChatGPT (GPT-5.4, GPT-5.5, and GPT-5.6, OpenAI, San Francisco, CA, USA) was used to (i) translate au-thor-written Chinese drafts into English and improve the grammar, wording, and log-ical flow of the English text; (ii) assist in searching and screening the literature, with every cited reference subsequently retrieved and checked against the original publica-tion by the authors; and (iii) assist in debugging and refactoring the Python analysis scripts, whose output was verified against manually computed results. The tool was not used to design the study, to acquire data, or to generate, alter, or interpret any ex-perimental data or results. The authors reviewed and edited all AI-assisted output and take full responsibility for the content of this manuscript.

Marker placement followed the upper-limb retro-reflective markers of the Plug-in Gait model [31]. Seven marker configurations were pre-defined according to the anatomical regions of the upper limb, the functional characteristics of the stroke kinetic chain and the requirements of the experimental application, following the natural segmentation of the human body. The number of markers and the number of model input channels of each configuration are listed in Table 2. The detailed anatomical placement of individual markers is presented in Supplementary Table S1.

### 3.3. Methodology

#### 3.3.1. Data Collection

Before the formal experiment, each participant completed a 10 min dynamic warm-up. After the warm-up and marker placement, participants performed the following 13 tennis strokes in sequence: the forehand and backhand flat, topspin, slice, volley, drop shot and overhead strokes, and the forehand serve. Forty valid trials were collected for the serve and 20 valid trials for each of the other 12 strokes. To prevent fatigue, a 7 min rest was provided between strokes. Except for the serve, which was self-tossed, all strokes were performed on balls fed by the ball machine. Therefore, before each new stroke type, participants were allowed to familiarize themselves with the trajectory and speed of the ball machine under that setting, and formal data collection began only after they had adapted. The 13 stroke classes are broadly consistent with the classification systems used in public tennis action datasets such as THETIS [26], which facilitates comparison with previous work.

#### 3.3.2. Data Preprocessing

After acquisition, the 3D motion-capture data were first labelled, checked and gap-filled in Vicon Nexus 2.16 to ensure that marker labelling was 100% complete for every trial. This guaranteed that the comparison among configurations was a paired comparison on the same set of samples, without differences in sample composition caused by unequal numbers of usable trials across configurations. Each stroke was then exported as a separate CSV file. Because acquisition was performed stroke by stroke, each CSV file corresponds to one complete stroke trial and covers the preparation phase before impact, the forward swing and impact phase, and the follow-through phase, so that the time window spans the complete stroke.

CSV files were read in Python and filtered with a fourth-order low-pass zero-phase (bidirectional) Butterworth filter with a cut-off frequency of 6 Hz, in order to avoid the phase lag introduced by unidirectional filtering [32]. Because the actual duration of a stroke differs across participants and stroke types, the number of raw frames contained in the CSV files was not identical. To construct multivariate time-series inputs of equal length, the complete movement trajectory of each trial was time-normalized and resampled to 128 time points. Specifically, the first and last frames of the raw trajectory were defined as the start and end of the normalized movement, 128 equally spaced target time positions were established between them, and one-dimensional linear interpolation was applied separately to the X, Y and Z coordinate series of each marker. Trials with more than 128 raw frames were compressed to 128 time points, and trials with fewer than 128 raw frames were interpolated to 128 time points. This procedure preserved the temporal order and the overall spatial shape of the movement trajectory while converting the absolute duration of different trials into a unified relative movement progression.

To facilitate the subsequent use of the data by machine-learning algorithms, and with reference to previous classifications of tennis strokes [2,10,25], a three-class task and a thirteen-class task were defined, in which the strokes were divided into three coarse-grained classes and 13 fine-grained classes. The three classes were the forehand class, the backhand class and the serve/overhead class. The 13 classes were forehand flat, forehand topspin, forehand slice, forehand overhead, forehand drop shot, forehand volley, backhand flat, backhand topspin, backhand slice, backhand overhead, backhand volley, backhand drop shot and serve.

A total of 11,200 trials were collected. All trials were inspected by researchers with a tennis-specific background, and a trial was excluded when there was an obvious execution error, when the required stroke type was not completed, when the target area was not effectively hit, or when the trajectory of a key marker could not be recovered. Finally, 10,133 valid trials were successfully processed and retained for the subsequent machine-learning analysis. This sample size is relatively large among comparable studies of tennis stroke recognition and helps to reduce the risk of overfitting under small-sample conditions [33].

#### 3.3.3. Implementation and Validation of the Machine-Learning Model

Because 3D motion-capture data have the typical characteristics of multivariate time series, MiniRocket was used for feature transformation and RidgeClassifierCV was used to discriminate stroke classes. Compared with deep models of similar purpose, the computational cost of MiniRocket is markedly lower: the original study reported that training and testing on the 109 datasets of the UCR archive could be completed in approximately 75 min on a single CPU core [30]. On the computing platform described above, 560 model trainings were performed in the present study (7 marker configurations × 2 classification tasks × 40 LOSO folds).

MiniRocketMultivariate from sktime was used to transform the 3D movement trajectories. For each marker, the position series in the X, Y and Z directions were extracted and the three coordinate components were treated as three mutually independent model input channels. Thus, one marker corresponds to three time-series channels, and the total number of input channels of a configuration equals the number of markers it contains multiplied by three.

MiniRocket applies a set of almost deterministic fixed convolutional kernels to the multichannel time series and uses proportion-of-positive-values (PPV) pooling to extract local trajectory-shape features at different temporal scales. The number of kernels was set to 10,000, the random seed was fixed at 42, and the parallelization parameter was set to n_jobs = −1 so that all available CPU cores were used where supported by the software version. Apart from the parameters described above, all other MiniRocket parameters were left at their package defaults. The same MiniRocket parameters were used for all classification tasks and all marker configurations; no manual, dataset-specific hyperparameter tuning was performed for any particular task or configuration.

The features extracted by MiniRocket were then fed into RidgeClassifierCV from scikit-learn for classification. This classifier uses L2 regularization to reduce the risk of overfitting caused by excessively large model coefficients. The regularization parameter α was selected automatically from 10 logarithmically spaced candidate values between 10^−3^ and 10^3^. Parameter selection was performed entirely within the current training data, and the external test data took no part in determining the regularization parameter. Apart from the candidate range of α, all other parameters of RidgeClassifierCV were left at their package defaults.

The cross-subject generalization performance of the model was evaluated with leave-one-subject-out (LOSO) cross-validation. Because 40 participants were included, each classification task and each marker configuration involved 40 outer validation folds. The folds were created in a fixed order of participant identifiers, and each participant was held out exactly once: all trials of that participant served as the test data of the fold, and the trials of the remaining 39 participants served as the training data.

Within each LOSO fold, the MiniRocket feature transformer was first fitted using only the data of the 39 training participants; the fitted transformer was then applied separately to the training set and to the test set of the held-out participant. The transformed training features were used to fit RidgeClassifierCV, and the regularization parameter α was selected within the training data. After classifier training, the class of every trial of the held-out participant was predicted. The data of the current test participant took no part in fitting the MiniRocket transformer, training the Ridge classifier or selecting the regularization parameter. After the 40 LOSO folds had been completed, the predictions for the held-out participants were pooled in turn to form overall predictions covering all participants, from which the evaluation metrics were computed.

Compared with a random trial-wise split into training and test sets, LOSO ensures that data from the same participant do not appear in both the training and the test set, thereby preventing the model from achieving high performance merely by exploiting individual-specific movement patterns, and providing a stricter evaluation of cross-participant generalization [34].

#### 3.3.4. Evaluation Metrics and Statistical Analysis

The evaluation metrics of the model were accuracy (Equation (1)), precision (Equation (2)), recall (Equation (3)), F1-score (Equation (4)), macro-averaged F1 (Macro-F1, Equation (5)), Weighted-F1 (Weighted-F1, Equation (6)) and the confusion matrix. Precision and recall were used mainly to compute Macro-F1. The F1-score is the harmonic mean of precision and recall and balances the two, preventing the model from favouring the majority class under class imbalance. To quantify the extent to which the simplified configurations retained the performance of the full-information configuration, a performance retention rate was further calculated as: retention rate = metric of the simplified configuration/metric of the full-information configuration × 100%.

(1) $$Accuracy=\frac{TP+TN}{TP+TN+FP+FN},$$

(2) $$Precision=\frac{TP}{TP+FP},$$

(3) $$Recall=\frac{TP}{TP+FN},$$

(4) $$F1-score=\frac{2\times Precision\times Recall}{Precision+Recall},$$

(5) $$Weighted-F1=\sum_{i=1}^{n}\frac{n_{i}}{N}F1_{i},$$

(6) $$Macro-F1=\frac{1}{n}\sum_{i=1}^{n}F1_{i}$$

where TP (true positive) is the number of trials correctly predicted as the given class, TN (true negative) is the number of trials correctly predicted as not belonging to the given class, FP (false positive) is the number of trials incorrectly predicted as that class, and FN (false negative) is the number of trials that belong to that class but were predicted as another class. All multiclass metrics were computed class by class in a one-vs-rest manner and then aggregated: n is the total number of classes (n = 3 for the three-class task and n = 13 for the thirteen-class task), F1_i_ is the F1-score of the i-th class, n_i_ is the number of trials of the i-th class, and N is the total number of trials. Macro-F1 is the arithmetic mean of the class-wise F1-scores (Equation (5)) and assigns equal weight to all classes, whereas Weighted-F1 is weighted by the proportion of trials of each class, n_i_/N (Equation (6)), and reflects overall performance under class imbalance.

**Figure 2:**
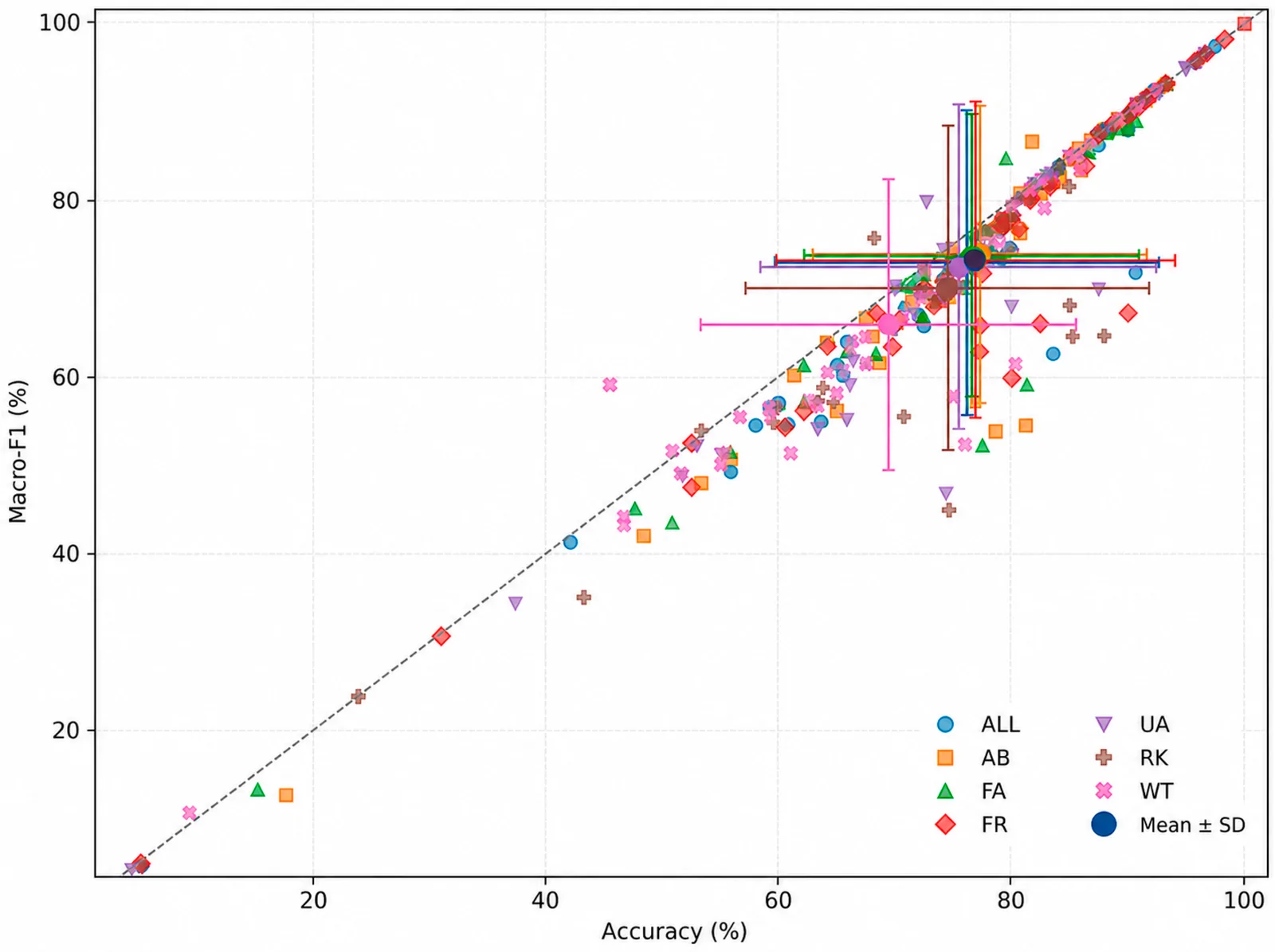
Relationship between accuracy and Macro-F1 in the thirteen-class task. Note: Each point represents the result of one participant; error bars denote the mean ± standard deviation; the dashed line is the y = x reference line.

The subject-level Macro-F1 values obtained from the 40-fold LOSO cross-validation were used as the unit of statistical analysis. Two forms of aggregation must be distinguished: the overall metrics (Table 3, Table 4 and Table 5) were computed once from the pooled predictions of the 40 folds and reflect the overall performance across all trials, whereas the subject-level metrics (Figure 2 and Table 6) were computed first within each held-out participant and then averaged arithmetically across the 40 participants, and were used as the unit of paired statistical testing. Because the two weighting schemes differ, the magnitude and even the direction of the difference between the same pair of configurations may differ between them; the form of aggregation used is therefore stated whenever values are reported. Continuous variables are described as mean ± standard deviation, and the normality of the paired differences between marker configurations was assessed with the Shapiro–Wilk test. When the paired differences did not satisfy the normality assumption, the Wilcoxon signed-rank test was used instead. For normally distributed paired data, paired-samples *t*-tests were used to compare Macro-F1 between marker configurations, and the mean difference, the 95% confidence interval and the Cohen’s dz effect size were reported. To control the accumulation of type I error caused by multiple pairwise comparisons, the *p*-values were corrected with the Holm method. All tests were two-tailed, the significance level was set at α = 0.05, and effect sizes were interpreted as negligible, small, medium and large for |dz| < 0.2, 0.2–0.5, 0.5–0.8 and ≥0.8, respectively.

## 4. Results

### 4.1. Recognition Results of the Marker Configurations in the Three-Class Task

The three-class task divided the 13 tennis strokes into the forehand class, the backhand class and the serve/overhead class. Table 3 lists the overall metrics and the class-level F1 values of the seven marker configurations in the three-class task. Overall, the accuracy of all seven configurations exceeded 98.50%, Macro-F1 exceeded 98.49% and Weighted-F1 values were all above 98.51%, indicating that the three coarse-grained classes could be distinguished stably by the model.

Accuracy ranged from 98.51% (WT) to 99.49% (ALL), Macro-F1 from 98.49% to 99.46% and Weighted-F1 ranged from 98.51% to 99.49%, with a range of less than one percentage point across configurations. At the class level, F1 was 98.68–99.67% for the forehand class, 98.26–99.50% for the backhand class and 98.35–99.22% for the serve/overhead class. Recognition performance in the three-class task was therefore close to saturation and could not differentiate the information efficiency of the marker configurations; the subsequent analyses therefore focused on the thirteen-class task.

### 4.2. Overall Performance of the Marker Configurations in the Thirteen-Class Task

Table 4 lists the overall recognition performance of the seven marker configurations in the thirteen-class task, that is, the average recognition performance across the 13 strokes. Compared with the three-class task, the overall metrics decreased markedly. Accuracy ranged from 74.11% to 80.15%, Macro-F1 from 72.07% to 78.76%, and Weighted-F1 from 74.00% to 80.13%. ALL achieved the highest accuracy, Macro-F1, and Weighted-F1, at 80.15%, 78.76%, and 80.13%, respectively. FR performed similarly to ALL, with corresponding values of 80.13%, 78.71%, and 80.11%, while RK reached 79.67%, 78.28%, and 79.67%, respectively.

The overall results of AB and FA were similar, with Macro-F1-scores of 75.61% and 75.32% and Weighted-F1-scores of 77.22% and 76.88%, respectively. WT and UA showed comparatively lower performance, with Macro-F1-scores of 73.07% and 72.07% and Weighted-F1-scores of 74.93% and 74.00%, respectively. Ranked by either Macro-F1 or Weighted-F1, the seven configurations followed the order ALL, FR, RK, AB, FA, WT, and UA, which was identical to the ranking obtained using accuracy. Overall, the Weighted-F1-scores were close to the corresponding accuracy values across all configurations, indicating a consistent performance pattern across the three evaluation metrics.

Figure 2 shows the relationship between subject-level accuracy and Macro-F1 in the thirteen-class task. Overall, the data points of all configurations were distributed mainly along the diagonal, indicating a high degree of agreement between accuracy and Macro-F1 in the present study. Because the two metrics showed essentially the same trends in the overall comparison, and because Macro-F1 treats all stroke classes equally and reduces the influence of class-size imbalance, Macro-F1 was used as the primary comparison metric in the subsequent results.

### 4.3. F1 Values of the Marker Configurations Across Stroke Classes in the Thirteen-Class Task

Table 5 lists the distribution of F1 values across stroke classes in the thirteen-class task, ordered by the mean F1 of the seven configurations. The differences among stroke classes were pronounced. The three strokes with the highest mean F1 were the serve, the forehand overhead and the backhand overhead, at 97.30%, 96.07% and 94.53%, respectively; the five strokes with the lowest mean F1 were the backhand volley, the backhand drop shot, the forehand slice, the backhand slice and the forehand flat, at 63.03%, 67.20%, 68.02%, 68.21% and 70.10%, respectively.

In terms of the best configuration for each stroke, FR achieved the highest F1 for five strokes, namely the backhand topspin, backhand flat, serve, forehand slice and forehand volley; RK achieved the highest F1 for three strokes, namely the backhand slice, backhand volley and backhand drop shot; ALL achieved the highest F1 for three strokes, namely the backhand overhead, forehand drop shot and forehand overhead; and AB achieved the highest F1 for two strokes, namely the forehand topspin and forehand flat.

In terms of the distribution of the worst configurations, UA produced the lowest F1 for seven strokes, WT for five strokes and RK for one stroke. In terms of the range of F1 across configurations for each stroke, the backhand slice, backhand topspin, forehand volley and forehand slice varied most, with ranges of 13.56, 13.10, 12.66 and 11.02 percentage points, respectively, whereas the serve and the forehand topspin varied least, with ranges of 1.54 and 3.56 percentage points, respectively.

### 4.4. Performance Retention Rate Relative to the Full-Information Configuration and Its Relationship with the Number of Channels

**Figure 3:**
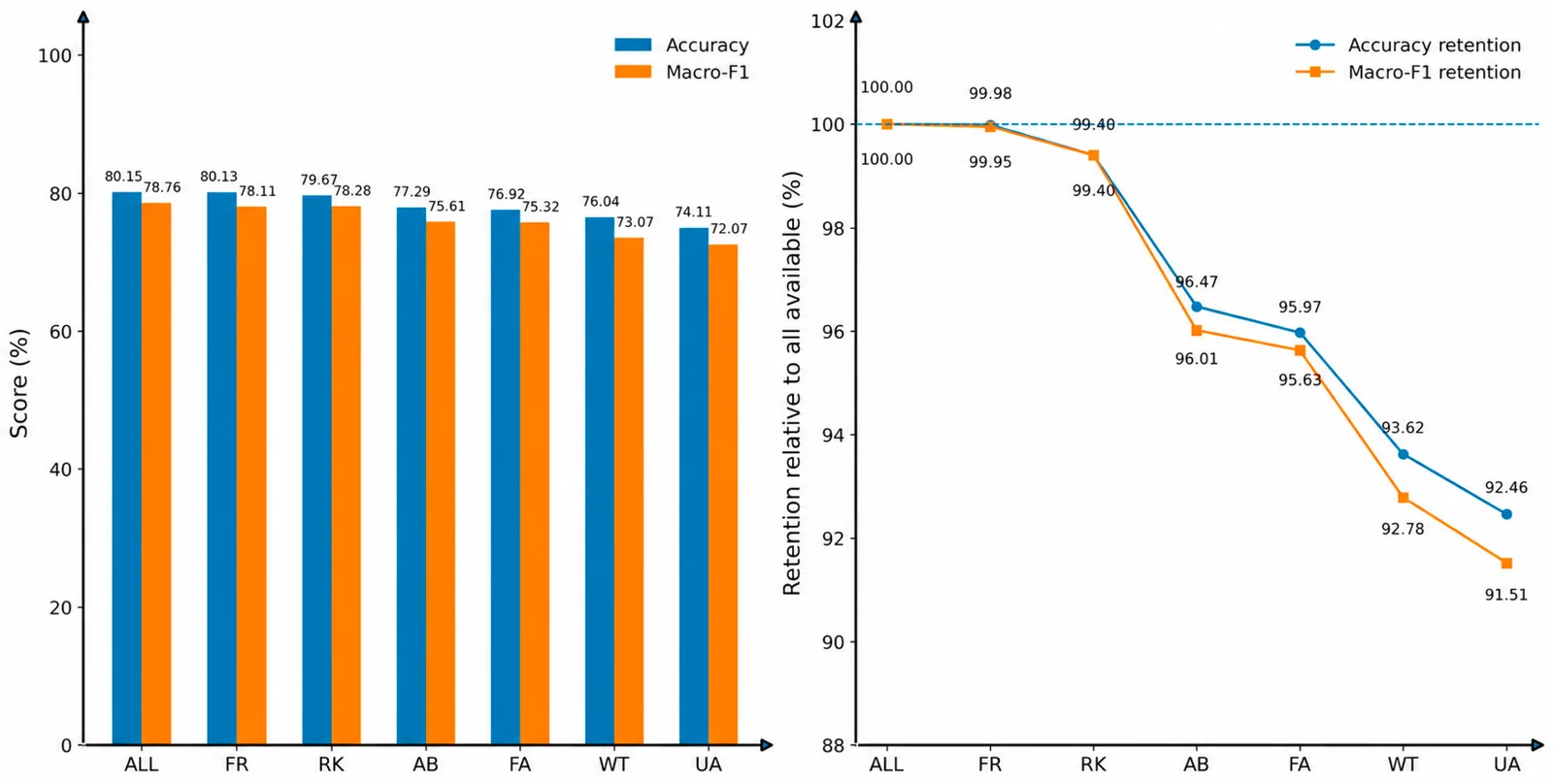
Overall accuracy, Macro-F1 and relative retention rates of the seven configurations in the thirteen-class task.

Using ALL as the reference, the performance retention rates of the simplified configurations in the thirteen-class task were calculated, and the results are shown in Figure 3. FR used 30 channels and retained 99.95% of the Macro-F1; RK used 15 channels and retained 99.40%; AB and FA retained 96.01% and 95.63%, respectively; and WT and UA were comparatively lower, at 92.78% and 91.51%.

**Figure 4:**
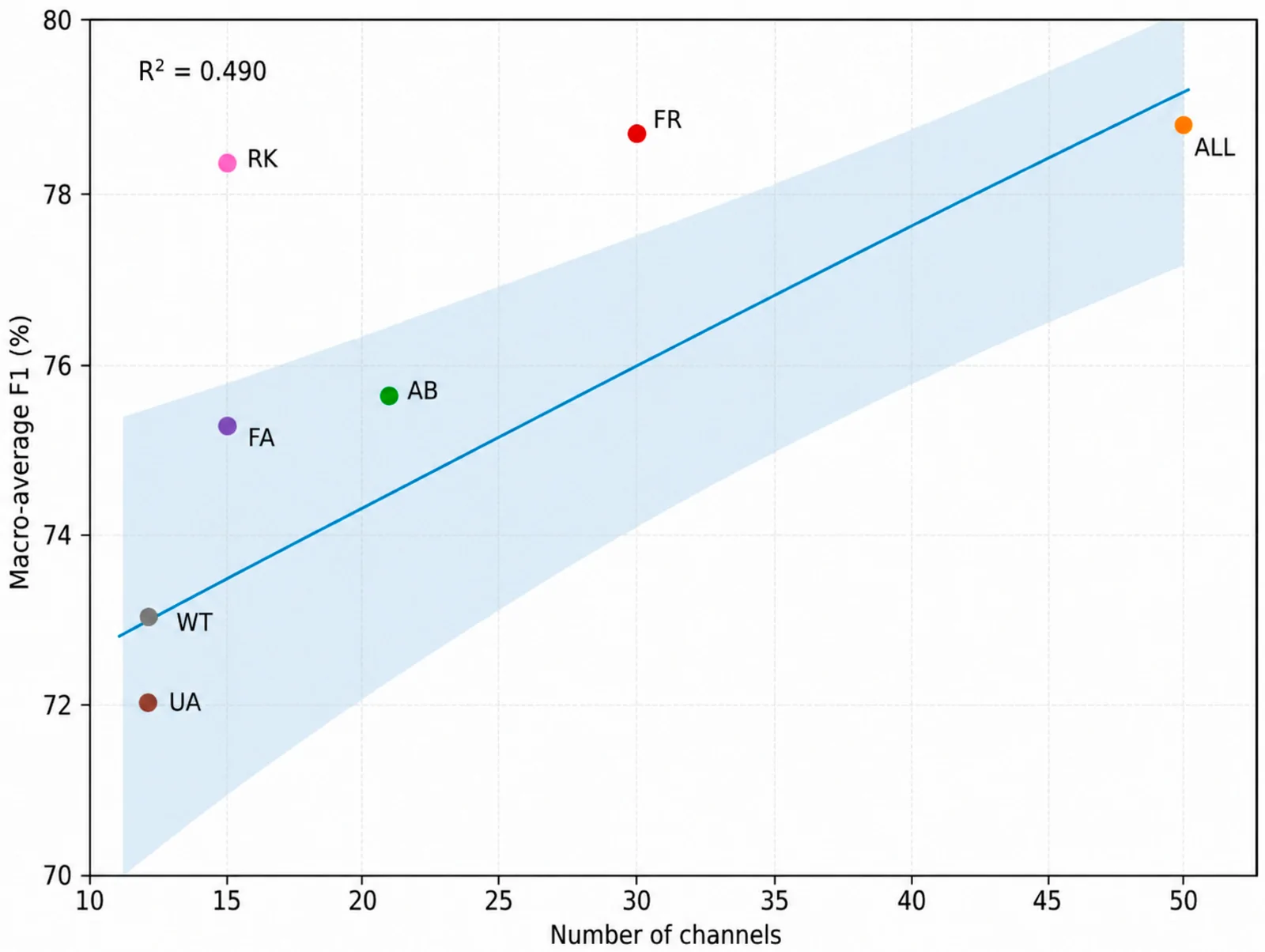
Scatter plot and linear regression of the number of channels against Macro-F1 for the seven configurations.

Figure 4 shows the linear relationship between the number of channels and Macro-F1. The seven configurations used 12–48 channels and achieved Macro-F1 values of 72.07–78.76%. Linear regression analysis indicated a moderate positive correlation between the number of channels and the macro-averaged F1 value (R^2^ = 0.49, r ≈ 0.70).

To further examine the differences among marker configurations, paired-samples *t*-tests were performed on the subject-level LOSO Macro-F1 results of the 40 participants, with Holm correction of the *p*-values to control the family-wise error rate and a significance level of α = 0.05. As shown in Table 6, the differences between ALL and FR, between ALL and RK, and between FR and RK did not reach statistical significance (all *p* > 0.05); the mean differences in these three comparisons were −0.15, −0.04 and 0.11 percentage points, the 95% confidence intervals all crossed zero, and the effect sizes were negligible (|dz| ≤ 0.05). By contrast, RK exceeded FA by 3.00 percentage points (dz = 0.51, medium effect), WT by 5.33 percentage points (dz = 0.73, medium effect) and UA by 6.30 percentage points (dz = 0.83, large effect), and these three comparisons remained statistically significant after Holm correction. The mean difference between RK and AB was 2.71 percentage points (dz = 0.40, small effect), with a corrected *p* = 0.088, which did not reach significance.

### 4.5. Confusion Matrices of the Marker Configurations in the Thirteen-Class Task

**Figure 5:**
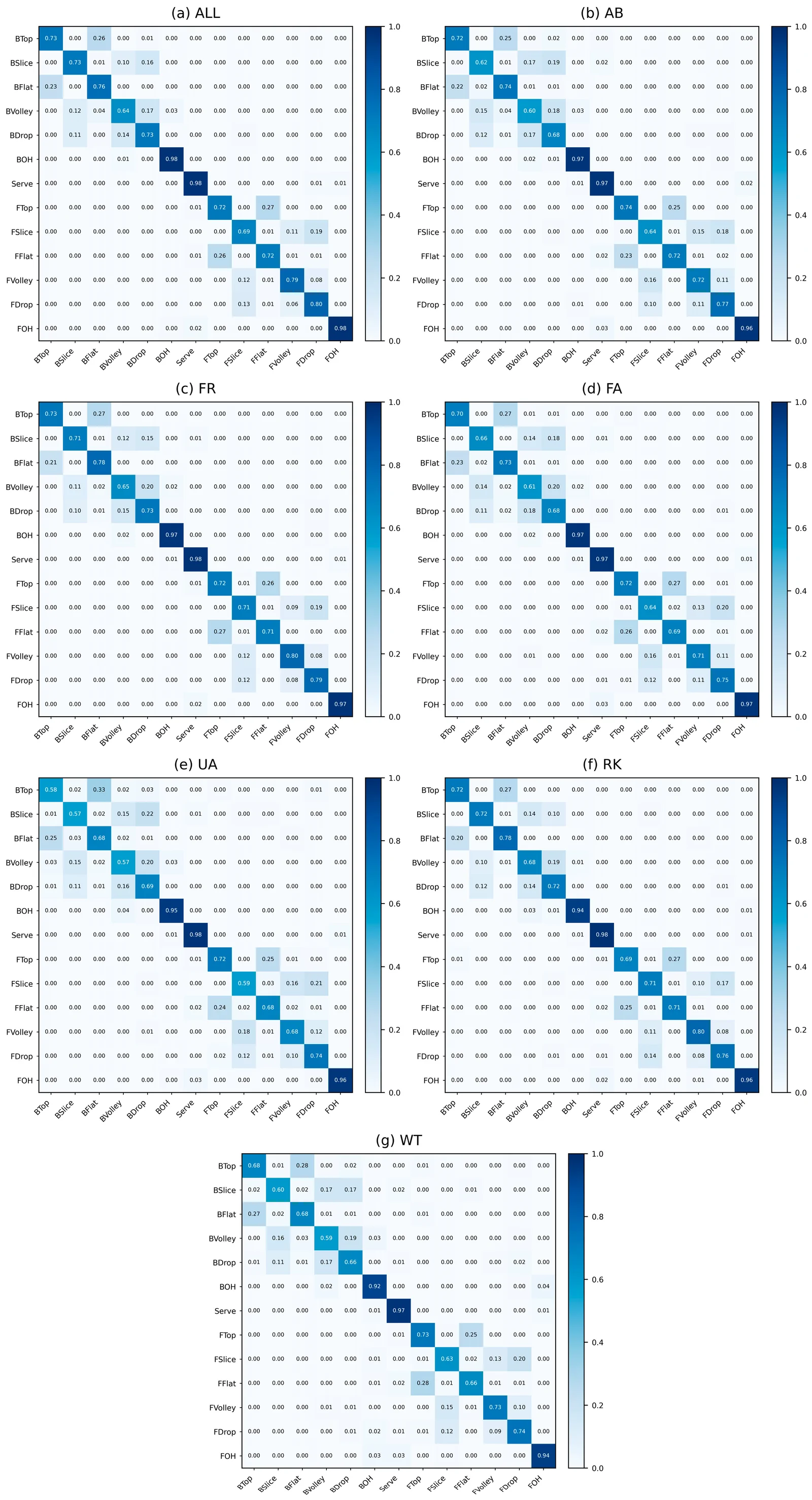
Normalized confusion matrices of the seven marker configurations in the thirteen-class task. Note: BTop, backhand topspin; BSlice, backhand slice; BFlat, backhand flat; BVolley, backhand volley; BDrop, backhand drop shot; BOH, backhand overhead; Serve, serve; FTop, forehand topspin; FSlice, forehand slice; FFlat, forehand flat; FVolley, forehand volley; FDrop, forehand drop shot; FOH, forehand overhead.

Figure 5 shows the normalized confusion matrices of the seven marker configurations in the thirteen-class task. Overall, the main values of all seven configurations were concentrated along the diagonal. The diagonal values of the serve, the backhand overhead, and the forehand overhead were high: across the seven configurations, the proportion of correctly classified serves ranged from 0.971 to 0.981, backhand overheads from 0.916 to 0.977, and forehand overheads from 0.938 to 0.979.

The diagonal values of some fine-grained baseline and net strokes were comparatively low. Ranked by the mean proportion of correct classification across the seven configurations, the backhand volley was lowest, with a mean of 0.620 and a range of 0.573–0.677, followed by the forehand slice, with a mean of 0.657 and a range of 0.585–0.710; the backhand slice had a mean of 0.658 and a range of 0.572–0.726, and the backhand topspin a mean of 0.693 and a range of 0.584–0.730.

The main misclassifications of the backhand strokes were concentrated among fine-grained techniques on the same side. Across the seven configurations, the backhand topspin was mainly misclassified as the backhand flat, with proportions of 25.16–32.66%, and the backhand flat was mainly misclassified as the backhand topspin, with proportions of 20.41–26.95%. The backhand volley was mainly misclassified as the backhand drop shot (16.97–20.30%), and the backhand drop shot as the backhand volley (14.26–17.65%). The main misclassifications of the forehand strokes were likewise concentrated within the same side: the forehand topspin was mainly misclassified as the forehand flat (24.81–27.31%) and the forehand flat as the forehand topspin (23.23–28.25%).

In terms of the comparison among configurations, ALL, FR and RK showed relatively high diagonal values for most strokes. UA showed low diagonal values for several fine-grained backhand strokes and for the forehand slice, for example, 0.584 for the backhand topspin, 0.572 for the backhand slice, 0.573 for the backhand volley, and 0.585 for the forehand slice. The confusion matrix results were generally consistent with the class-level F1 results: the serve and the overhead strokes were classified correctly at a high rate, whereas considerable confusion occurred among same-side fine-grained techniques such as flat–topspin, volley–drop shot, and slice–drop shot.

## 5. Discussion

Using 3D motion capture and the MiniRocket time-series classification model, this study compared the performance of seven upper-limb and racket marker configurations in tennis stroke recognition, with the aim of examining how different upper-limb and racket marker configurations affect recognition performance. The results showed that (i) in the coarse-grained three-class task all configurations achieved high recognition performance, and (ii) in the fine-grained thirteen-class task ALL achieved the best overall recognition performance, but FR and RK maintained recognition performance close to that of ALL while markedly reducing the number of channels. Taken together with the class-level F1 values, the confusion matrices, the retention rates and the paired tests, these findings indicate that the discriminative information for tennis stroke recognition is not evenly distributed across all markers and that it is more strongly represented in the forearm-wrist-racket chain.

In the fine-grained thirteen-class task, the accuracy and Macro-F1 of the full-information configuration were 80.15% and 78.76%, respectively (Table 4). Compared with the three representative public datasets THETIS, Tennis-MoCap and 3DTennisDS, the present study differs clearly and adds value in terms of experimental design, coverage of stroke classes and the strategy of comparing marker configurations. An Inception model applied to the full 12 classes of THETIS reported an accuracy of 84.10% under LOSO validation [3,4]. Although that dataset contains 12 stroke classes and 55 participants, it lacks racket information and can hardly capture the subtle movement relationship between the distal upper limb and the implement. The representative studies on Tennis-MoCap and 3DTennisDS both selected 4 stroke classes and used ST-GCN with a random 60%/20%/20% split, obtaining accuracies of 77.26% and 81.85%, respectively [6,8]. These values cannot be interpreted directly as evidence that one dataset, acquisition system or classification model is superior to another, because the studies differ markedly in participant level, number of stroke classes, data modality, sample size and validation strategy. The present study achieved performance comparable to that reported for the three public datasets while using fewer markers, and on this basis analyzed the different configurations, providing more interpretable experimental evidence for the optimization of marker placement in 3D motion-capture experiments and for sensor placement in wearable devices. The results are analyzed in detail below.

The three-class task mainly distinguishes the forehand class, the backhand class, and the serve/overhead class. These three classes differ clearly in swing side, racket movement direction, impact height, and movement amplitude, so the model could readily establish stable class boundaries. As shown in Table 3, the accuracy of all seven configurations exceeded 98.50%, and Macro-F1 exceeded 98.49%, with only small numerical differences among configurations. This result is also consistent with previous work: most studies of tennis action recognition have addressed coarse-grained classes such as the serve, forehand and backhand and have generally reported high recognition performance [11,12,13,14]. A similar trend was observed here with 3D motion-capture data, indicating that combining 3D motion capture with machine-learning computation can stably recognize the major categories of tennis strokes.

The thirteen-class task further requires the model to distinguish specific techniques within the same side, including flat, topspin, slice, volley, drop shot and overhead strokes. Table 4 shows that the Macro-F1 of the seven configurations fell to 72.07–78.76%, markedly lower than in the three-class task, indicating a higher trajectory similarity and a greater classification difficulty among fine-grained techniques. Research indicates that these fine-grained techniques often share similar preparation postures and parts of the swing path, and that the differences lie mainly in racket-face control, the direction of racket-head velocity, the vertical component and the follow-through within a short period around impact [35]. Specifically, FR maintained a Macro-F1 close to that of ALL after the number of channels had been reduced, indicating that the forearm, wrist, elbow and racket jointly constitute a region of high information density for tennis stroke classification. This echoes the conclusions of Skublewska-Paszkowska et al. [2], who reported that adding racket markers improved recognition performance but that the contribution of racket information did not reach statistical significance (*p* > 0.05). The tennis stroke is a typical proximal-to-distal kinetic-chain movement: the trunk and the upper arm provide the basic power, whereas the forearm and the wrist are directly involved in racket-face orientation, racket-head path and control at the instant of impact [16]. Studies of forehand technique have found differences in upper-limb muscle activation and swing control between flat and topspin forehands [36], and studies of the double-handed backhand have likewise shown that forearm, wrist and elbow kinematics are closely related to the execution of the topspin backhand [19]. With respect to the relationship between the number of channels and Macro-F1, RK and FA both used 15 channels, yet RK was significantly better than FA in Macro-F1. This result indicates that model performance depends not only on the number of channels but also on whether the markers are located at the key sites where discriminative information is concentrated. One of the main differences between topspin and flat strokes lies in the vertical component of racket-head motion and the direction of the follow-through, whereas slices, volleys and drop shots depend more on racket-face control and short-amplitude swings [17]; these features are likely to be preserved more directly by the racket markers. UA and WT showed relatively low recognition performance in the thirteen-class task; together with the linear regression result in Figure 4 (R^2^ = 0.49), which indicates that the number of channels is not the dominant factor determining performance, this suggests that the position information of a single body segment has limited explanatory power for fine-grained technique recognition. The relatively poor performance of UA and WT can be attributed to the fact that the upper arm mainly reflects proximal shoulder and elbow motion and large-amplitude upper-limb swings, whereas the wrist reflects only part of the kinematic information of the forearm; neither adequately reflects joint changes when distinguishing techniques within the same side. The data distribution further shows that when a configuration includes markers along the forearm–wrist–racket chain, performance already approaches that of the best configuration ALL (≥78.0%), and adding further body or upper-arm markers brings no substantial gain in performance. Therefore, in the design of resource-constrained wearable devices, priority should be given to covering the key segments rather than simply pursuing a larger number of channels.

The overall performance of the different marker configurations shows that providing more channels does not necessarily bring a proportional improvement in performance. Table 4 and Figure 4 show that FR and RK retained 99.95% and 99.40% of the Macro-F1 of ALL, respectively, while the number of input channels decreased from 48 to 30 and 15. The participant-level comparisons in Table 6 further show that the mean difference between ALL and FR was only −0.15 percentage points and that between ALL and RK only −0.04 percentage points. These differences did not reach statistical significance and the standardized effect sizes were close to zero, indicating that under the present sample and experimental conditions the numerical performance of the three configurations was very similar. By contrast, RK exceeded FA by 3.00 percentage points with a medium effect size (dz = 0.51), and exceeded WT and UA by 5.33 and 6.30 percentage points with effect sizes of 0.73 and 0.83, respectively, all of which remained statistically significant after Holm correction (Table 6). These results indicate that the discriminative information is more clearly expressed along the forearm–wrist–racket kinetic chain.

In a comparison of accelerometer positions, Woll et al. likewise found that multi-sensor configurations were not consistently superior to the best single sensor position and that model performance depended more on whether the sensor was located at a body site relevant to the target task [18]. Table 5 presents a comparison of the extreme F1 values of the seven marker configurations across stroke classes. FR achieved the highest F1 for five strokes, RK for three strokes, and ALL and AB for three and two strokes, respectively; at the same time, UA showed the lowest performance for seven strokes and WT for five strokes. In particular, the backhand slice, the backhand volley and the backhand drop shot achieved their highest F1 values under the RK configuration, indicating that the differences among these strokes may be reflected more directly in the short-amplitude trajectory of the racket and in racket-face control. The forehand topspin and the forehand flat performed best under the AB configuration, indicating that some strokes may still benefit from more complete arm-movement information. These results suggest that the degree to which different strokes depend on different segment configurations is not uniform and that a correspondence exists between stroke type and segment configuration. This finding is also consistent with the conclusion of tennis IMU research that the sensor mounting position affects classification performance [25]. Therefore, within a framework combining 3D motion capture and machine learning, the segment configuration is itself an important factor influencing classification performance, and this result provides an important reference for future research on channel selection, local-region weighting and attention-based modelling.

The class-level F1 values and the normalized confusion matrices provide a more concrete explanation of the difficulty of the thirteen-class task. Both Table 5 and Figure 5 show that the serve and the overhead strokes were the easiest to recognize, whereas confusion occurred more readily among same-side flat, topspin, slice, volley and drop shot strokes. The serve and the overhead are clearly overhead strokes and differ from baseline and net strokes in impact height, racket-head path, the amplitude of upper-limb elevation and movement rhythm, and therefore have clearer spatiotemporal patterns. Misclassification between flat and topspin strokes was concentrated within the same side, which is related to their highly similar technical structure. The two usually share a similar preparation posture and forward-swing direction, and the differences lie mainly in the racket-face angle near impact, the low-to-high swing amplitude and the follow-through trajectory; these differences may occur only within a short time window. Volleys and drop shots also have small swing amplitudes and short movement durations, so the local trajectory differences are relatively subtle [37].

From an applied perspective, the present results provide a basis for optimizing marker placement in future 3D motion-capture experiments in tennis. If the aim is coarse-grained action recognition, training feedback or rapid movement screening, marker configurations related to the forearm already show high information efficiency; if the aim is to recognize the 13 specific stroke techniques, configurations that include the forearm–wrist–racket chain are more information-efficient. Specifically, RK may offer a more favourable balance between recognition performance and acquisition complexity, particularly in scenarios where rapid deployment, reduced marker occlusion, participant comfort or computational efficiency are prioritized. Compared with the full marker set, FR achieves a good balance between reducing the number of markers, shortening preparation time and economic cost, reducing the data-cleaning workload and maintaining model performance. For other types of sensors, however, such as IMUs or accelerometers, mounting them on the racket is problematic because their mass and volume may significantly alter the racket’s weight, centre of mass and other inertial properties. In contrast, FR may remain preferable in controlled laboratory studies that require detailed forearm kinematics or the complete forearm–racket movement relationship, and is more compatible with the practical demands of sensor placement. The final choice between FR and RK should depend on the error tolerance of the target application scenario, particularly for stroke classes such as volleys and drop shots that remain comparatively difficult to distinguish. Wrist information was already highly usable in the three-class task, but was inferior to FR and RK in the thirteen-class task. This result suggests that a wrist-worn device alone is better suited to recording training load, counting strokes and recognizing coarse categories such as forehand/backhand and serve. Further distinguishing specific technique types may require the combination of a racket-mounted sensor or a distal forearm signal.

## 6. Limitations and Future Perspectives

This study still has several limitations. First, it was based on laboratory-based 3D motion-capture technology, which, compared with previous studies using portable sensors, lacks certain ecological validity in real-game contexts. Second, although the study employed LOSO cross-validation to enhance the rigour of cross-subject evaluation, the sample size, the number of stroke types, and the distributions of participants’ body morphologies, playing styles, and skill levels may still affect the stability of the results. Third, the study used MiniRocket as the primary machine learning model, and it remains unclear whether the conclusions drawn from the same marker configurations can be generalized to other model architectures or to other sports. Fourth, tennis is a whole-body sport, yet this study only examined the impact of upper-limb markers on recognition accuracy, without discussing the effects of trunk and lower-limb markers on machine learning recognition performance.

In future work, with larger samples and more comprehensive marker sets, attention mechanisms, channel selection networks, or other time-series models could be introduced to further examine the robustness of different marker configurations, to explore the sensitivity of various models to information from key body segments, and to attempt the removal of redundant markers in order to identify optimized configurations. This line of research could even be extended to areas such as stroke quality assessment, injury risk evaluation, and the detection of sports fatigue. Meanwhile, recent research has focused on bidirectional multi-scale feature aggregation, adaptive feature selection, and joint channel–spatial attention mechanisms [38,39]. Although these artificial intelligence algorithms have not yet been applied to tennis action recognition, their feature fusion strategies may provide useful architectural inspiration for modelling multi-scale temporal patterns and interactions among motion-capture channels.

## 7. Conclusions

This study shows that a multivariate time-series model based on MiniRocket can effectively recognize tennis strokes from 3D motion-capture data. In the coarse-grained three-class task, the model showed a high recognition ability for the forehand class, the backhand class, and the serve/overhead class; in the thirteen-class task, the model still achieved good overall performance, although the recognition difficulty differed markedly among specific strokes. With MiniRocket fixed as the classification method, the full-information configuration achieved the highest macro-averaged F1 value in the thirteen-class task, but its advantage over the forearm + racket configuration was minimal; the forearm + racket and racket configurations maintained performance close to that of the full-information configuration while markedly reducing the number of channels, indicating that the discriminative information for tennis stroke recognition is more strongly represented in the forearm-wrist-racket chain. These findings can provide a basis for the optimization of marker placement in future tennis motion-capture experiments, for the construction of action-recognition models, and for sensor placement in wearable devices.

## Figures and Tables

**Table 1:** Comparison of representative public tennis action recognition datasets.

Dataset	Acquisition System	Scale	Information Collected	Representative Model and Validation	Reported Performance
THETIS	Microsoft Kinect; RGB, depth, 2D/3D	55 participants; 12 classes; 8374 videos	human skeleton and video; no racket model	Inception + three-layer LSTM; leave-one-subject-out validation	84.10% (accuracy)
Tennis-MoCap	6 OptiTrack Flex V100 cameras; 100 Hz; BVH	17 participants; 6 classes; 106 motion files	34 body markers; no racket model	ST-GCN; 4-class subset; 60/20/20 random split × 20	77.26% (accuracy)
3DTennisDS	8 Vicon cameras; 100 Hz; C3D	10 professional players; 4 classes; 769 trials	39 body markers; 7 racket markers	ST-GCN; 4 classes; 60/20/20 random split × 20	81.85% (accuracy)

**Table 2:** The seven marker configurations and their numbers of channels.

Full Name	Abbreviation	Number of Markers	Number of Channels	Markers Included
Full-Information Group	ALL	16	48	SHOUPA, ELB, MELB, FRM, WRA, WRB, Watch, Rocket
Full-Arm Group	AB	7	21	SHO, UPA, ELB, MELB, FRM, WRA, WRB
Forearm Group	FA	5	15	ELB, MELB, FRM, WRA, WRB
Forearm + Racket Group	FR	10	30	ELB, MELB, FRM, WRA, WRB, Watch
Upper-Arm Group	UA	4	12	SHO, UPA, ELB, MELB
Racket Group	RK	5	15	Rocket
Watch Group	WT	4	12	Watch

**Table 3:** Overall performance and class-level F1 values of the seven marker configurations in the three-class task.

Configuration	Accuracy (%)	Macro-F1 (%)	Weighted-F1 (%)	Forehand F1 (%)	Backhand F1 (%)	Serve/Overhead F1 (%)
ALL	99.49	99.46	99.49	99.67	99.50	99.22
AB	99.22	99.20	99.22	99.40	99.10	99.11
FR	99.38	99.35	99.38	99.56	99.47	99.03
FA	99.31	99.28	99.31	99.54	99.28	99.03
UA	98.56	98.55	98.56	98.79	98.26	98.59
RK	99.04	99.02	99.04	99.20	99.04	98.84
WT	98.51	98.49	98.51	98.68	98.45	98.35

**Table 4:** Overall recognition performance of the seven configurations in the thirteen-class task.

Configuration	Channels	Accuracy (%)	Macro-F1 (%)	Weighted-F1 (%)
ALL	48	80.15	78.76	80.13
AB	21	77.29	75.61	77.22
FR	30	80.13	78.71	80.11
FA	15	76.92	75.32	76.88
UA	12	74.11	72.07	74.00
RK	15	79.67	78.28	79.67
WT	12	75.04	73.07	74.93

**Table 5:** Comparison of the extreme F1 values of the seven marker configurations across tennis stroke classes.

Stroke Class	Mean F1 Across 7 Configs (%)	Best Config.	Best F1 (%)	Worst Config.	Worst F1 (%)
Serve	97.30	FR	98.06	WT	96.52
Forehand overhead	96.07	ALL	97.62	WT	92.80
Backhand overhead	94.53	ALL	96.90	WT	89.60
Forehand volley	75.12	FR	80.37	UA	67.71
Forehand drop shot	73.02	ALL	76.00	UA	69.73
Forehand topspin	72.26	AB	74.45	RK	70.89
Backhand flat	72.00	FR	75.69	UA	66.67
Backhand topspin	70.81	FR	74.46	UA	61.36
Forehand flat	70.10	AB	72.06	WT	67.62
Backhand slice	68.21	RK	74.01	UA	60.45
Forehand slice	68.02	FR	72.30	UA	61.28
Backhand drop shot	67.20	RK	70.78	WT	63.96
Backhand volley	63.03	RK	67.47	UA	58.20

**Table 6:** Pairwise comparisons of Macro-F1 between key configurations in the thirteen-class task.

Comparison	Metric	Mean Difference (Percentage Points)	95% CI	*p* (Paired *t*-Test)	Holm-Corrected *p*	Cohen’s dz
ALL vs. FR	Macro-F1	−0.15	−1.05 to 0.74	0.733	1.000	−0.05
ALL vs. RK	Macro-F1	−0.04	−1.27 to 1.18	0.942	1.000	−0.01
FR vs. RK	Macro-F1	0.11	−1.04 to 1.25	0.850	1.000	0.03
RK vs. AB	Macro-F1	2.71	0.56 to 4.86	0.015	0.088	0.40
RK vs. FA	Macro-F1	3.00	1.13 to 4.87	0.002	0.019	0.51
RK vs. WT	Macro-F1	5.33	2.98 to 7.68	<0.001	<0.001	0.73
RK vs. UA	Macro-F1	6.30	3.89 to 8.71	<0.001	<0.001	0.83

CI, confidence interval. *p*-values were corrected with the Holm method.

## Data Availability

The data presented in this study are available from the corresponding authors upon reasonable request.

## Supplementary Materials

The following supporting information can be downloaded at: https://www.mdpi.com/article/10.3390/s26165081/s1, Table S1. Specific anatomical locations of each marker.
