# Supplementary material for: Effects of Different Marker Set Configurations on Tennis Stroke Recognition Performance: A Three-Dimensional Kinematic Study Based on MiniRocket
Source: Sensors (Basel). 2026 Aug 11;26(16):5081. doi: 10.3390/s26165081 (PMC13517808; doi:10.3390/s26165081)
Supplement: Supplementary file 1 [file sensors-26-05081-s001.zip › sensors-4463791-supplementary.pdf]

Table S1. Specific anatomical locations of each marker.

Marker label	Definition	Anatomical placement
**SHO**	**Shoulder**	**Over the acromioclavicular joint**
**UPA**	**Upper arm**	**On the upper lateral one-third of the upper arm**
**ELB**	**Lateral elbow**	**Over the lateral epicondyle of the humerus**
**MELB**	**Medial elbow**	**Over the medial epicondyle of the humerus**
**FRM**	**Forearm**	**On the lower lateral one-third of the forearm**
**WRA**	**Wrist marker A**	**On the radial side of a bar attached to the dorsal aspect of the wrist, as close to the wrist joint center as possible**
**WRB**	**Wrist marker B**	**On the ulnar side of a bar attached to the dorsal aspect of the wrist, as close to the wrist joint center as possible**

Note: The WT configuration consisted of a rigid watch-mounted cluster containing four reflective markers, which was secured to the surface of the wristwatch. The RK configuration consisted of five reflective markers distributed on the racket. Both marker clusters were designed with asymmetric spatial arrangements to enable reliable identification of their anterior and posterior orientations during motion capture. The specific marker layouts were custom-designed and remained unchanged throughout all data-collection sessions.
